# Comparative Analyses of Chloroplast Genomes for Parasitic Species of Santalales in the Light of Two Newly Sequenced Species, *Taxillus nigrans* and *Scurrula parasitica*

**DOI:** 10.3390/genes14030560

**Published:** 2023-02-23

**Authors:** Ximing Yue, Ning Miao, Zilu Fan, Kangshan Mao

**Affiliations:** Key Laboratory of Bio-Resource and Eco-Environment, Ministry of Education, College of Life Sciences, Sichuan University, Chengdu 610064, China

**Keywords:** *Taxillus nigrans*, *Scurrula parasitica*, chloroplast genome, phylogenetic, Santalales, parasitic species

## Abstract

When a flowering plant species changes its life history from self-supply to parasite, its chloroplast genomes may have experienced functional physical reduction, and gene loss. Most species of Santalales are hemiparasitic and few studies focus on comparing the chloroplast genomes of the species from this order. In this study, we collected and compared chloroplast genomes of 12 species of Santalales and sequenced the chloroplast genomes of *Taxillus nigrans* and *Scurrula parasitica* for the first time. The chloroplast genomes for these species showed typical quadripartite structural organization. Phylogenetic analysis suggested that these 12 species of Santalales clustered into three clades: *Viscum* (4 spp.) and *Osyris* (1 sp.) in the Santalaceae and *Champereia* (1 sp.) in the Opiliaceae formed one clade, while *Taxillus* (3 spp.) and *Scurrula* (1 sp.) in the Loranthaceae and *Schoepfia* (1 sp.) in the Schoepfiaceae formed another clade. *Erythropalum* (1 sp.), in the Erythropalaceae, appeared as a third, most distant, clade within the Santalales. In addition, both *Viscum* and *Taxillus* are monophyletic, and *Scurrula* is sister to *Taxillus*. A comparative analysis of the chloroplast genome showed differences in genome size and the loss of genes, such as the *ndh* genes, *infA* genes, partial ribosomal genes, and tRNA genes. The 12 species were classified into six categories by the loss, order, and structure of genes in the chloroplast genome. Each of the five genera (*Viscum*, *Osyris*, *Champereia*, *Schoepfia*, and *Erythropalum*) represented an independent category, while the three *Taxillus* species and *Scurrula* were classified into a sixth category. Although we found that different genes were lost in various categories, most genes related to photosynthesis were retained in the 12 species. Hence, the genetic information accorded with observations that they are hemiparasitic species. Our comparative genomic analyses can provide a new case for the chloroplast genome evolution of parasitic species.

## 1. Introduction

Parasitic plants obtain all or most nutrients and water from their host plants and are often divided into hemiparasitic and holoparasitic species [1,2]. Hemiparasitic species can obtain nutrients and water from their host species and can produce organic matter by conducting photosynthesis. Holoparasitic species, however, cannot conduct photosynthesis as their leaves are usually degraded into squamous and there is insufficient chlorophyll [3]. On the tree of life, most parasitic plant species are nestled in different clades of autotrophic plant species, suggesting that they most likely experienced a transformation in life history from autotroph to parasite [4]. During this process, many morphological changes may have occurred, including the reduction of roots into haustoria [5], the decrease of photosynthetic tissues, etc. At the same time, at the genetic level, the chloroplast genomes of parasitic plant species experience a functional and physical reduction, gene loss, etc. [6,7,8]. Since parasitic plants use their haustoria to penetrate their host plants’ tissues and uptake nutrients, their ability to produce carbohydrates via photosynthesis may have degraded, their photosynthetic tissues may have decreased, and their chloroplast genomes may have degraded [3,9]. The chloroplast genomes of parasitic plants have attracted the attention of many researchers [10], but there are few comparative analyses of the chloroplast genomes of different families of hemiparasitic plants in Santalales.

Chloroplast is a key organelle of green plants used for photosynthesis [11], during which organic matter is generated and energy is stored [12]. Chloroplast is thought to be an endosymbiotic organelle and it has an independent and conserved genome [13]. The chloroplast genome is presented as multiple copies of covalent, closed, and conserved circular double-stranded DNA that is single-parentally inherited. However, many studies have shown that it can also be biparentally inherited [14,15,16]. The specific double-stranded circular chloroplast DNA molecule ranges in length from 115 to 165 kb. Usually it consists of four parts: a short single copy (SSC), a long single copy (LSC), and two inverted repeats (IR) [17]. The chloroplast genome contains around 110 genes in most plant species, but several genes are absent in the chloroplast genomes of some plant species [9]. It is prevalent in parasitic plant species whose chloroplast genomes have lost many photosynthetic genes [18]. For many parasitic plant species, their photosynthesis function is reduced, or they cannot photosynthesize, leading to changes in their chloroplast genomes [13,19,20]. Therefore, compared to photosynthetic plant species, the genetic content of the chloroplast genome of parasitic plant species is significantly reduced [13]. These genes are primarily associated with photosynthesis, such as photorespiration genes [21], protein coding genes [22], ribosomal protein genes [23], and transfer RNA (tRNA) genes [22,23]. A few genes remain as pseudogenes. Genes associated with genetic mechanisms are usually retained [19,20,24]. The first holoparasitic plant species sequenced chloroplast genome was *Epifagus virginiana*, whose genome is only 70 kb and contains only 42 protein-encoding genes and 13 tRNA genes [25]. This species has lost all photosynthesis and energy-producing genes; only a few of those genes remain as pseudogenes, and the entire chloroplast cannot conduct photosynthesis [13].

Santalales is an order of woody flowering plant species. Its members are primarily found in subtropical regions, most of which are hemiparasitic plant species that can produce organic matter through photosynthesis. Still, those plants need to obtain water and minerals via haustoria used to penetrate the stems or roots of their host plants. In this study, we sequenced and assembled the chloroplast genomes of two species in the family Loranthaceae in the order Santalales: *T. nigrans* and *S. parasitica*. We compiled ten species of Santalales whose chloroplast genomes were available on NCBI Genbank before April 2018. We compared the size, structure, presence, and absence of genes of the chloroplast genomes in the 12 species of Santalales and constructed the maximum likelihood tree of those species. A previous study of *T. nigrans* and *S. parasitica* found that the EST-SSR markers developed based on transcriptomic data of *T. nigrans* were successfully identified in *S. parasitica* individuals, suggesting a close relationship between those two species [26]. In this study, we aimed: (a) to figure out the variation of size and assembly of the chloroplast genomes of the 12 species of Santalales, (b) to infer the evolution of the chloroplast genome structure of the 12 species, and (c) to infer the phylogenetic relationship of the 12 species within the order Santalales. Our comparative genomic analyses can provide a new case for the chloroplast genome evolution of parasitic species.

## 2. Materials and Methods

### 2.1. DNA Sequencing and Chloroplast Genome Assembly

The experimental samples we collected were the fresh leaves of *T. nigrans* and *S. parasitica*, that parasitize on *Platanus acerifolia* and *Ligustrum lucidum*, respectively. The samples were collected at Sichuan University. We immediately froze those leaves in a liquid nitrogen tank and sent the frozen leaves to the Novogene Company for DNA extraction and genome sequencing. The modified CTAB method [27] was used to extract the total DNA of the leaf samples and Nanodrop was applied to detect DNA purity (OD 260/280 ratio). Sequencing was performed using the Illumina High-throughput Sequencing Platform (HiSeq/MiSeq). Finally, we obtained raw reads of 6.73 G and 6.85 G from *T. nigrans* and *S. parasitica* samples, respectively, and their GC contents were 42.3% and 41.6%, respectively.

We used the Trimmomatic v0.36 [28] to filter the raw reads and obtain high-quality clean reads. The BWA-MEM V0.7.12 [29] was used to compare the clean reads using *T. chinensis* as the reference chloroplast genome (Genbank: NC_036306.1). The read sequence was mapped to the corresponding reference genome. We used NOVOPlasty v2.6.3 [30] and Velvet v1.2.07 [31] to assemble and splice chloroplast genomes. We spliced contigs into scaffold sequences and then used them to assemble the chloroplast genome.

### 2.2. Gene Annotation and Sequence Analyses

We used Geneious v11.0.3 [32] to check and then save the assembly results as fasta files. We used Plann V1.1 [33] to annotate the *T. nigrans* and *S. parasitica* chloroplast by referring to the effects of *T. chinensis* chloroplast genome annotation. We then used Geneious v8.1.4 [32] and Sequin v15.10 to correct the annotation results. The final chloroplast genomes sequence of *T. nigrans* and *S. parasitica* was submitted to GenBank. The chloroplast gene map was obtained using the online program Organellar Genome DRAW (OGDRAW) v1.2 (http://ogdraw.mpimp-golm.mpg.de/ (accessed on 12 April 2018). To analyze the characteristics of the variations in synonymous codon usage, we used MEGA6 [34] to obtain the relative synonymous codon usage values (RSCU) and codon usage by neglecting the influence of amino acid composition.

### 2.3. Genome Comparison

The genomes of the 12 species of Santalales were compared using the online program mVISTA (http://genome.lbl.gov/vista/mvista/submit.shtml (accessed on 15 April 2018)). In addition to *T. nigrans* and *S. parasitica*, chloroplast genomes data for another 10 species of Santalales were downloaded from the NCBI database, including five Santalaceae species: *Viscum album* (NC_028012.1)*, V. crassulae* (NC_027959.1)*, V. coloratum* (NC_035414.1)*, V. minimum* (NC_027829.1), *Osyris alba* (NC_027960.1); two Loranthaceae species: *T. chinensis* (NC_036306.1), *T. sutchuenensis* (NC_036307.1), Schoepfiaceae: *Schoepfia jasminodora* (NC_034228.1); Olacaceae: *Erythropalum scandens* (NC_036759.1) and Opiliaceae: *Champereia manillana * (NC_034931.1). We labeled the gene exon, intron sites, and transcriptional direction for *T. nigrans* and *S. parasitica*, and submitted that information to the online program mVISTA (http://genome.lbl.gov/vista/mvista/submit.shtml(accessed on 15 April 2018). We ran mVISTA twice: once using *T. nigrans* as the reference, and again using *S. parasitica* as the reference.

### 2.4. Phylogenetic Analysis

Phylogenetic trees were constructed based on the chloroplast genomes of 12 species of Santalales to analyze their phylogenetic relationships. We used MAFFT V7.158 [35] and MEGA v6.0 [34] to extract and align the amino acid sequences of the proteins encoded by their common genes. An ML tree was constructed by the RAxML V8.2.11 software [36] based on the PROTGAMMAJTT model. The outgroups consist of two holophytes (*Pvrola. rotundifolia* (KU833271.1) and *Vaccinium. macrocarpon* (NC019616.1). Then we used FigTree v1.4.3 to check the results.

## 3. Results

### 3.1. Characteristics of T. nigrans and S. parasitica Chloroplast Genomes

**Figure 1:**
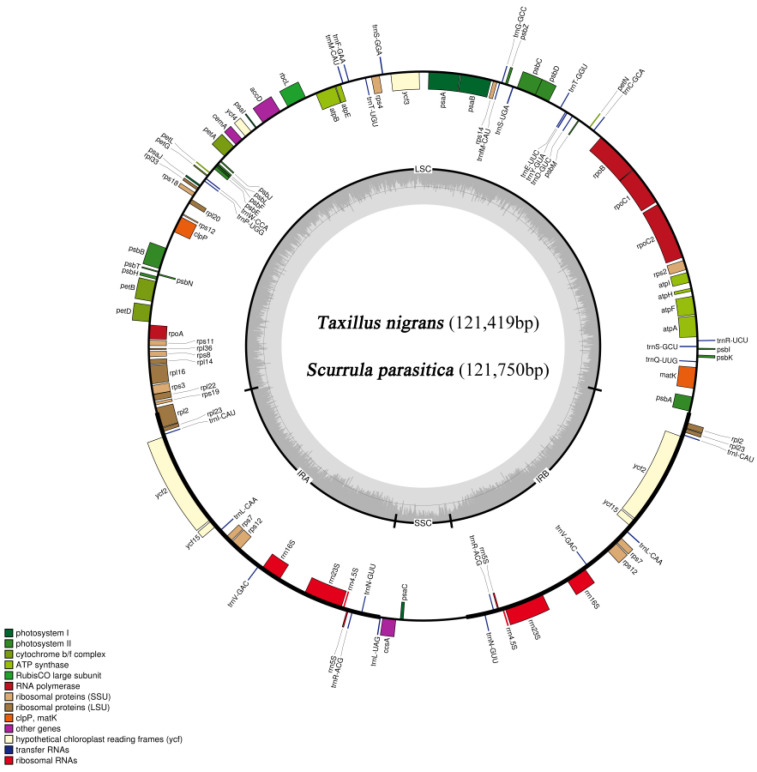
Gene map of *T. nigrans* and *S. parasitica* chloroplast genome (the darker gray in the inner circle corresponds to GC content, while the lighter gray corresponds to AT content).

The chloroplast genomes of *T. nigrans* and *S. parasitica* are circular molecules that retained the typical structure (Figure 1). The lengths of the chloroplast genomes of those two species were 121,419 bp and 121,750 bp, respectively. Both chloroplast genomes comprised IR regions (*T. nigrans*, 22,569 bp; *S. parasitica*, 22,687 bp) that were separated by the LSC region (*T. nigrans*, 70,181 bp; *S. parasitica*, 70,270 bp) and the SSC region (*T. nigrans*, 6100 bp; *S. parasitica*, 6106 bp) (Figure 1). The GC contents of the *T. nigrans* and *S. parasitica* chloroplast DNA were 37.4% and 37.2%, respectively. These were unevenly distributed throughout their chloroplast genomes (Table 1). A total of 106 genes were annotated in both the *T. nigrans* and *S. parasitica*, including four pseudogenes (Table 2), eight rRNA genes, 28 tRNA genes, and 66 protein-coding genes.

In *T. nigrans*, 3993 codons (9.9%) were encoded for leucine, while 581 (1.4%) were encoded for tryptophan. Similarly, in *S. parasitica*, 4027 codons (9.9%) were encoded for leucine and 524 (1.3%) were encoded for tryptophan. In both species, leucine was the most prevalent and tryptophan was the least prevalent of these amino acids. The complete chloroplast genome sequence of *T. nigrans* and *S. parasitica* has been deposited in GenBank under accession numbers MH095982 and MH101514, respectively.

### 3.2. Comparative Chloroplast Genomic Analysis

A comparative analysis of the chloroplast genomes of the 12 species of Santalales (Table 3) demonstrated that the lengths of the genomes varied from 118 kb to 156 kb. The length of the *S. jasminodora* chloroplast genome was the shortest and the length of the *E. scandens* chloroplast genome was the longest. *E. scandens* had the largest LSC (84,799 bp) and SSC (18,567 bp) of the 12 species. However, *E. scandens* also had the smallest proportion of LSC and the largest proportion of SSC of the 12 species. The length of IR varied from 22 k to 28 kbp. *S. jasminodora* had the smallest IR, much shorter than the IRs of the other 11 species, but its LSC had the largest proportion of chloroplast genomes. *C. manillana* had the largest IR (28,075 bp) and the largest proportion of chloroplast genomes.

By comparing the lengths of LSC, SSC, and IR (Table 3), the chloroplast genomes from the 12 species can be classified into six categories: *Viscum*, *Osyris*, *Champereia*, *Schoepfia*, and *Erythropalum.* Each represented an independent category, while the three *Taxillus* species and *Scurrula* represented a sixth category. The three *Taxillus* species and *Scurrula* had a minimal chloroplast genome size and composition differences and the smallest proportion of the SSC. Meanwhile, the lengths of the chloroplast genome, LSC, SSC, and IR of the four *Viscum* species were similar in gene numbers, position, and mVista analysis. Yet, their total number of genes, number of protein-coding genes, and tRNA genes were different. When comparing the above two categories, the three *Taxillus* species, one *Scurrula* species, and the four *Viscum* species all showed slight differences. However, among all 12 species, the remaining four categories (*Champereia*, *Erythropalum*, *Osyris*, and *Schoepfia*) all showed apparent differences, especially concerning the lengths of the chloroplast genome, SSC, and IR (Table 3).

**Figure 2:**
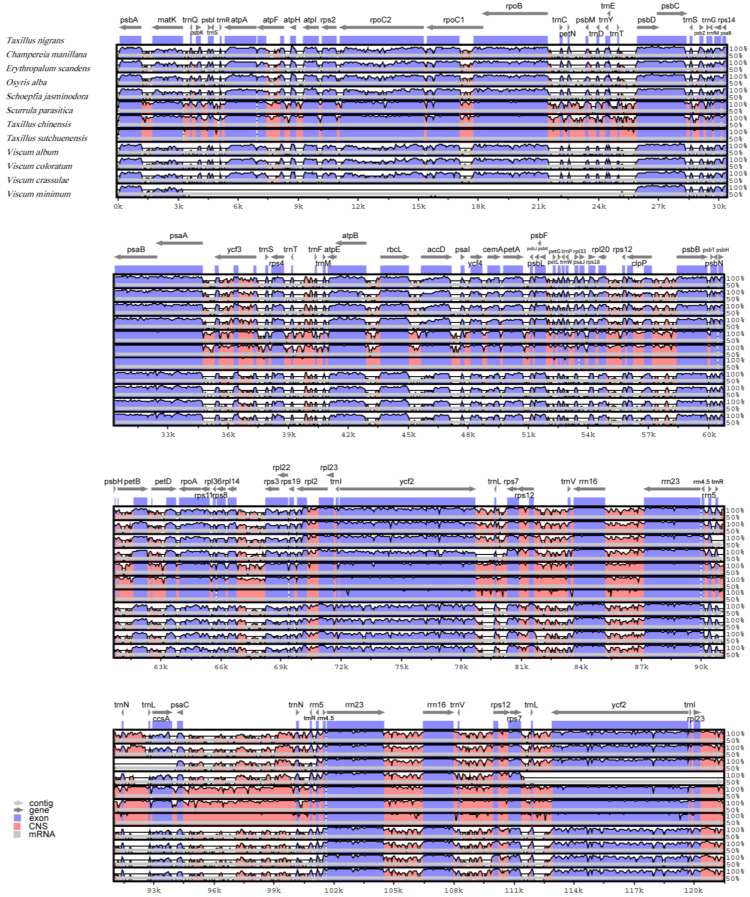
Comparison of the chloroplast genomes of the 12 species, using *T. nigrans* as a reference in the mVISTA program. (Grey arrows and thick black lines above the alignment indicate the orientation of genes. A cut-off of 70% identity was used for the plots. The Y-scale axis represents the percent identity within 50%–100%. Genomic regions are color-coded as either protein-coding exons, rRNAs, tRNAs, or conserved non-coding sequences (CNS), the same as below).

**Figure 3:**
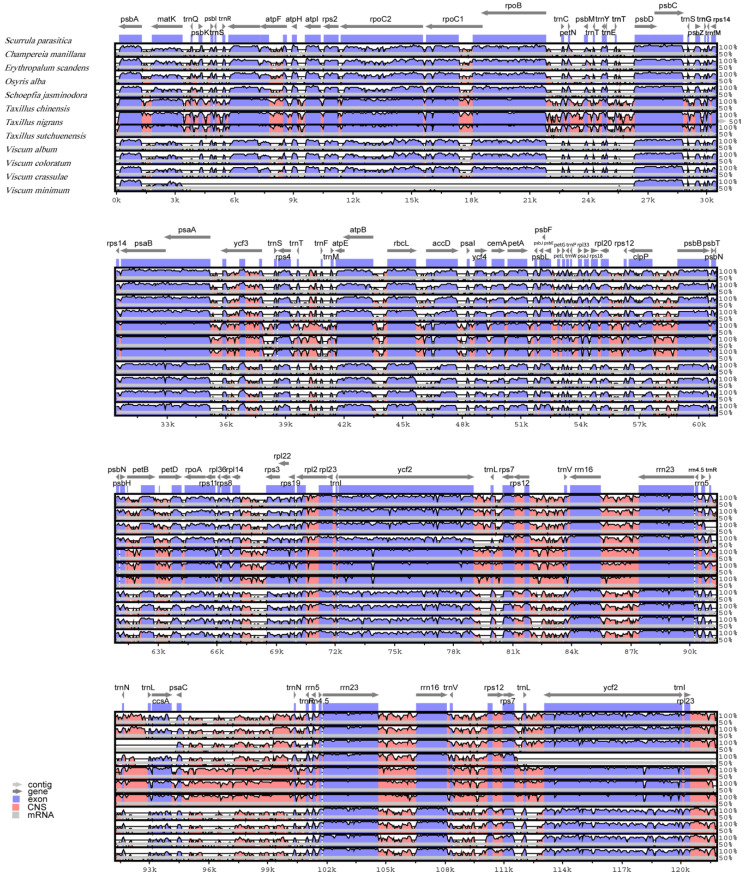
Comparison of the chloroplast genomes of the 12 species, using *S. parasitica* as a reference in mVISTA program.

Overall, among the chloroplast genomes of the 12 species, the coding areas were more conservative than the non-coding areas. The IR had a lower divergence than the LSC and SSC. Eight rRNA genes did not have relatively large indels and were highly conserved. TRNA genes and protein-coding genes, such as *rpoC2* and *ycf2*, had large indels. The difference between the chloroplast genomes of *T. nigrans* and *T. sutchuenensis* was the smallest. The chloroplast genomes of the three species of *Taxillus* and *S. parasitica* were very similar to each other (Figure 2 and Figure 3).

In the chloroplast genomes of the 12 species, eight rRNA genes, several *rps* genes (*rps2*, *3*, *4*, *7*, *8*, *11*, *12*, *14*, *18*, and *19*), *rpl* genes (*rpl2*, *14*, *20*, *22*, *23*, and *36*), and *trn* (*E* and *fM*) genes were all present and were relatively conservative. The DNA sequences from gene *trnT-GGU* to gene *trnQ-UUG* in *V. minimum* were found to be in a reverse direction compared to the other 11 species. The *trnR-ACG* and *trnN-GUU* genes were absent in *O. alba*; at the same time, the DNA sequences of the *ccsA* plus *trnL-UAG* genes were found to be in a reverse direction when compared to the other 11 species.

By comparing and analyzing the chloroplast genomes of the 12 species of Santalales, we found that, in the three *Taxillus* species and the one *Scurrula* species, some genes were missing, including several NAD(P)H dehydrogenase complex subunits (*ndh* gene), four ribosomal protein genes (*rpl32*, *rps15*, *rps16,* and *rps33*), one *ycf* gene (*ycf1*), and the initiation factor gene (*infA*). Ten tRNA genes (*trnL-UAA*, *trnI-GAU*, *trnK-UUU*, *trnP-GGG*, *trnP-TGG*, *trnH-GUG*, *trnQ-UUG*, *trnG-UCC*, *trnV-UAC,* and *trnA-UGC*) were also missing in the chloroplast genomes of these four species. Two ribosomal protein genes (*rpl16* and *rpl2*) and the repeat gene *ycf15* degenerated into pseudogenes in the same four species as their gene coding regions were interrupted by deletions, insertions, or internal stop codons. The pseudogene *rpl2* was in the IRb region. The 12 species of Santalales retained several photosystem genes (*psaA*, *B*, *C*, *I*, *J*, *ycf*3, 4 and *psbA*, *B*, *C*, *D*, *E*, *F*, *H*, *I*, *J*, *K*, *L*, *M*, *N*, *T*, *Z*), *pet* genes (*petA*, *B*, *D*, *G*, *L*, *N*), *atp* genes (*atpA*, *B*, *E*, *F*, *H*, *I*), *rpo* genes (*rpoA*, *B*, *C1*, *C2*), and the *rbcL* gene.

**Figure 4:**
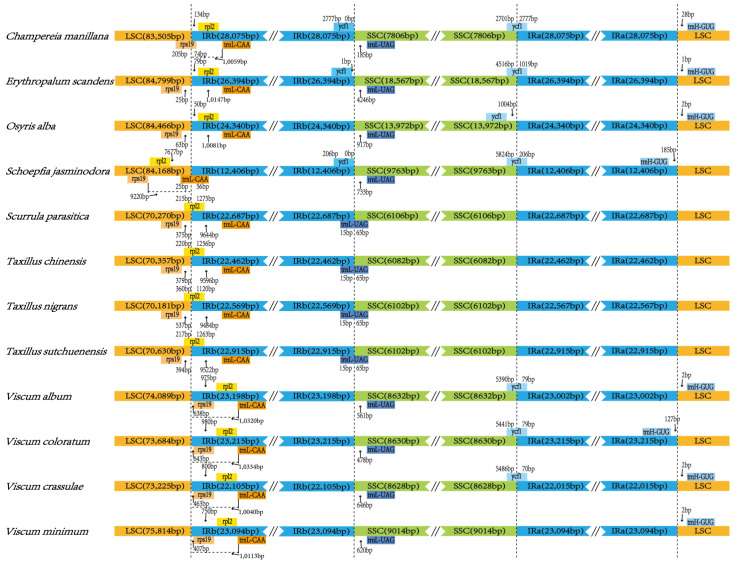
Comparison of border distance between adjacent genes and junctions of the LSC, SSC, and two IR regions among the chloroplast genomes of the 12 species of Santalales (The figure shows relative changes at or near the IR/SSC borders).

At the LSC/IRb junction, *rpl2* genes locate here and extend to LSC regions with different lengths for the four Loranthaceae species (*T. sutchuenensis*, 217 bp; *T. nigrans*, 360 bp; *T. chinensis*, 220 bp; and *S. parasitica*, 215 bp). In *S. jasminodora*, the *rpl2* gene was in the LSC region, 7677 bp away from the IRb region. The *rpl2* genes in *E. scandens*, *C. manillana*, and the five species of Santalaceae (*O. alba* and the 4 *Viscum* species) were in the IRb region (*E. scandens*, 79 bp; *C. manillana*, 134 bp; *O. alba*, 50 bp; *V. album*, 975 bp *V. coloratum*, 980 bp; *V. crassulae*, 800 bp; and *V. minimum*, 750 bp). In *C. manillana*, the junction of LSC/IRb was the *rps19* gene, extending 205 bp to the LSC region. In the *O. alba*, *S. Jasminodora*, and the 4 Loranthaceae species, the *rps19* gene was in the LSC region (*E. scandens*, 25 bp; *O. alba*, 63 bp; *S. jasminodora*, 9220 bp; *S. parasitica*, 375 bp; *T. chinensis*, 379 bp; *T. nigrans*, 537 bp; and *T. sutchuenensis*, 394 bp). Among the 4 *Viscum* species, the *rps19* gene was in the IRb region (*V. album*, 638 bp; *V. coloratum*, 643 bp; *V. crassulae*, 463 bp; *V. minimum*, 407 bp). In *S. jasminodora*, the *trnL-CAA* gene was located at the junction of LSC/IRb and extended 26 bp into the LSC region. The *trnL-CAA* gene was in the IRb region for the other 11 species (*V. album*, 10,320 bp; *V. coloratum*, 10,334 bp; *V. crassulae*, 10,040 bp; *V. minimum*, 10,113 bp; *O. alba*, 10,081 bp; *C. manillana*, 10,059 bp; *T. chinensis*, 9596 bp; *T. nigrans*, 9484 bp; *T. sutchuenensis*, 9522 bp; *S. parasitica*, 9644 bp; and *E. scandens*, 10,147 bp). In the IRb/SSC junction, the 4 Loranthaceae species were *trnL-UAG* genes, extending 65 bp to the SSC region. The *trnL-UAG* genes of the other eight species are all located in the SSC region (*C. manillana*, 185 bp; *E. scandens*, 4246 bp; *O. alba*, 917 bp; *S. jasminodora*, 733 bp; *V. album*, 561 bp; *V. coloratum*, 478 bp; *V. crassulae*, 646 bp; *V. minimum*, 620 bp). For *C. manillana*, *E. scandens*, and *O. alba*, the *ycf1* gene was in the IRb region, 0 bp, 1 bp, and 0 bp away from the junction; the other nine species have lost the *ycf1* gene near IRb/SSC junction. For *C. manillana*, *E. scandens*, *S. jasminodora*, *V. album*, *V. coloratum*, and *V. crassulae*, the *ycf1* gene were located at the SSC/IRa junction, which extends to SSC regions 2701 bp, 4516 bp, 5284 bp, 5390 bp, 5441 bp, and 5486 bp, respectively. For *O. alba*, the *ycf1* gene was in the SSC region, 1004 bp away from the junction. In other species, the *ycf1* gene has been lost from the chloroplast genome. All species had no genes at the IRa/LSC junction. The *trnH-GUG* gene was in the LSC region in *C. manillana*, *E. scandens*, *O. alba*, *V. album*, *V. crassulae*, *V. minimum* species, 28 bp, one bp, two bp, two bp, two bp, and two bp from junctions, respectively. Both in *S. jasminodora* and *V. coloratum* are in the IRa area, 185 bp and 127 bp from the junction (Figure 4). The *trnH-GUG* gene was lost from the chloroplast genomes in the other four species.

### 3.3. Phylogenetic Analysis

**Figure 5:**
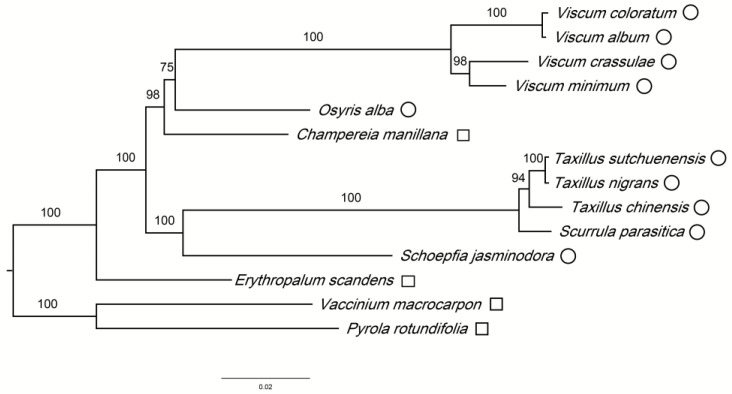
Maximum likelihood phylogenetic tree of Santalales based on 39 protein-coding genes. (Hemiparasitic plants are labeled with circles (○), and autotrophic plants are marked with white squares (□)).

The chloroplast genome can provide essential data for evolution, taxonomy, and phylogenetic studies. 11 out of the 12 nodes in the maximum likelihood tree received 94% to 100% bootstrap support (Figure 5). Phylogenetic analysis revealed that 11 out of the 12 species of Santalales clustered into two highly supported clades. One clade includes *Viscum* (4 spp.), *Osyris* (Santalaceae), and *Champereia* (Opiliaceae), while the other clade includes *Taxillus* (3 spp.), *Scurrula* (Loranthaceae) and *Schoepfia* (Schoepfiaceae). In addition, a third clade is the most distant clade among the Santalales, and only includes *Erythropalum* (Erythropalaceae). While the monophyly of *Viscum* was strongly sustained, the monophyly of Santalaceae received moderate bootstrap support (75%), as indicated by the apparent sister relationship between *Viscum* (Visceae) and *Osyris* (Santaleae). In this way, the monophyly of Santalaceae deserves closer research.

## 4. Discussion

At the third codon position, it prefers A and T. Also, the preference for A and T appears at the stop codons. We found that the usage of A-ending and U-ending is generally excessive. Other than *trnL-CAA* and *trnS-GGA,* all types of synonymous codons (RSCU > 1) prefer to end with A or U (Table 4). Commonly, it prefers A and T in plant chloroplast genomes at the third codon position [37]. This universal law can differentiate chloroplast DNA from mitochondrial and nuclear DNA [37].

Advances in phylogenetic studies indicate that the chloroplast genome’s evolution involves nucleotide substitutions and changes in genomic structure [37,38]. Examples of the latter include the loss of genes and introns. Previous studies have shown that introns significantly regulate gene expression and selective splicing, enhancing exogenous gene expression at particular sites in plants at specific times. It has been noted that introns can significantly stabilize transcription in some eukaryotes [13,39]. The chloroplast genomes of *T. nigrans* and *S. parasitica* contained seven intron-existing genes, including *atpF*, *rpoC1*, *ycf3*, *rps12*, *petB*, *petD*, and *rpl2* genes (Table 5). Among them, the *ycf3* gene, located in the LSC, contained two introns and three exons. The *rps12* gene was dedicated to trans-splicing, with the 5′ exon in the LSC and the 3′ exon in the IR. Comparative analysis revealed that the size of the *rps12* gene was also decreased due to the loss of cis-spliced introns. However, the ability of the *rpsl2* gene to code and express could remain intact; none of the gene’s coding region has been degraded due to its reduced length or frameshift mutation [40].

The loss of genes often occurs during the life cycle transition from autotrophy to parasitism in plants [13,41]. Overall, all 12 species of Santalales in this study retained some photosystem genes, indicating that these species have a relatively complete photosynthetic capacity despite some loss of photosynthesis-related genes. The comparative analysis of chloroplast genomes showed that both *T. nigrans* and *S. parasitica* lost the *ndh* gene and *infA* gene in that chloroplast genome, which is consistent with general models of plastome degradation [42]. The *ndh* gene plays an essential role in plant photoautotrophy. Its expression marks significant plant transition evolution [43]. However, cases where the *ndh* gene was missing or degenerated into pseudogenes have been found in the chloroplast genomes of many parasitic plant species [13,37,38,44,45]. By comparing gene content, plastome structure, and selection pressure, Li, *et al.* [45] found that hemiparasitism accelerates the pseudogenization and loss of the plastid *ndh* gene of Orobanchaceae plant species. Genetic changes in parasitic plants of Santalales are usually characterized by pseudogenization or loss of the *ndh* complex gene [42].

Among the 12 species of Santalales in this study, the *ndhA* gene located in the SSC of *S. jasminodora* and the repeat gene *ndhB* located in the IR of the species *V. minimum* all degenerated into pseudogenes. More importantly, however, except for *E. scandens* (an autotrophic plant), all *ndh* genes were found missing in the other nine species of Santalales, include autotrophic plant *C. manillana*. These results indicate that the photosynthetic capacity of these species gradually degraded during the evolution to heterotrophy, which may also reflect the increased host dependence of these species. As for *E. scandens*, the *ndh* gene in its chloroplast genome is more intact than the other 11 Santalales species. The *ndhJ*, *K*, and *C* genes located in the LSC and the repeat gene *ndhB* located in the IR, the *ndhF*, *D*, *E*, *G*, *I*, *A*, and *H* in the SSC of the *E. scandens* chloroplast genome were retained and the chloroplast genome is the longest. These results demonstrated that the photosynthetic capacity of *E. scandens* was stronger than that of hemiparasites such as *T. nigrans* and *S. parasitica*, and that it is less dependent on its host. Our findings suggest that the lifestyle transition of parasitic plants is accompanied by the relaxation of chloroplast gene purifying selection [7,42]. However, the chloroplast gene of hemiparasitic plants evolution is comparatively conserved in the hemiparasitic plants. Our study supports the idea that hemiparasitic plants still reserve the ability of photosynthesis and can produce organic matter by conducting photosynthesis.

## 5. Conclusions

We used high-throughput sequencing technology to sequence the chloroplast genome sequences of two hemiparasitic species: *T. nigrans* and *S. parasitica*. The sequencing, assembly, annotation, and comparative analysis showed that the *T. nigrans* chloroplast genome was 121,419 bp and the *S. parasitica* chloroplast genome was 121,750 bp. A total of 106 genes of *T. nigrans* and *S. parasitica* were annotated, including 66 protein-coding genes, 28 tRNA genes, eight rRNA genes, and four pseudogenes. In the comparison of the chloroplast genomes of the 12 species of Santalales, *E. scandens* chloroplast DNA was the largest and the *S. jasminodora* chloroplast DNA was the smallest. All *ndh* genes associated with NAD(P)H dehydrogenase have become pseudogenes or have been completely lost, while some tRNA genes have been lost, a few ribosomal protein genes and *ycf* genes have been degraded, and the loss of these genes has significantly reduced the size of SSC and LSC of *T. nigrans* and *S. parasitica* chloroplast DNA. Phylogenetic analysis showed that 11 of the 12 species were clustered into two clades with high bootstrap support. In agreement with phylogenetic analyses, the loss of genes, the order of genes, and the structure of genes in the chloroplast genomes of these species can be assigned to six categories: *Viscum* (4 spp.), *Osyris* (Santalaceae), and *Champereia* (Opiliaceae) formed one clade, and *Taxillus* (3 spp.), *Scurrula* (Loranthaceae), and *Schoepfia* (Schoepfiaceae) formed another clade. *Erythropalum* (Erythropalaceae) was the most distant clade within the Santalales. Our phylogenetic relationship among the families of the 12 species of Santalales, based on the chloroplast genomes, is consistent with recently reported phylogenetic trees (e.g., Angiosperm Phylogeny Website, Version 14, 2017). Although different genes are lost in various categories, most genes related to photosynthesis are retained in the 12 species. Hence, the genetic information from chloroplast genomes accorded with observations that they are hemiparasitic plants. This study will provide information for further research about the chloroplast DNA evolution and phylogenetic and molecular ecology of the family Santalales, and our comparative genomic analyses provide a new case for the chloroplast genome evolution of parasitic plants.

## Figures and Tables

**Table 1:** Elemental composition in the *T. nigrans* and *S*. *parasitica* chloroplast genomes.

Species		T(U) (%)	C (%)	A (%)	G (%)	Length (bp)
*T. nigrans*	LSC	33.4	18.0	31.8	16.8	70,181
	SSC	41.2	13.3	32.5	13.0	6100
	IRa	28.8	20.6	28.3	22.3	22,569
	IRb	28.3	22.3	28.7	20.7	22,569
	Total	32.0	19.0	30.6	18.4	121,419
*S. parasitica*	LSC	33.5	17.7	31.9	16.7	70,270
	SSC	41.5	12.8	32.6	13.1	6106
	IRa	28.8	20.5	28.3	22.4	22,687
	IRb	28.3	22.4	28.8	20.5	22,687
	Total	32.1	18.9	30.7	18.3	121,750

**Table 2:** List of genes in the *T. nigrans* and S. *parasitica* chloroplast genomes.

Genes		
1	Photosystem I	*psaA, B, C, I, J, ycf3, 4*
2	Photosystem II	*psbA, B, C, D, E, F, H, I, J, K, L, M, N, T, Z*
3	Cytochrome b6/f	*petA, B, D, G, L, N*
4	ATP synthase	*atpA, B, E, F, H, I*
5	Rubisco	*rbcL*
6	RNA polymerase	*rpoA, B, C1, C2*
7	Small subunit ribosomal proteins	*rps2, 3, 4, 7, 8, 11, 12, 14, 18, 19*
8	Large subunit ribosomal proteins	*rpl2, 14, 20, 22, 23, 33, 36*
9	Other proteins	*accD, ccsA, cemA, clpP, matK*
10	Proteins of unknown function	*ycf2*
11	Ribosomal RNAs	*rrn4.5S, rrn5S, rrn16S, rrn23S*
12	Transfer RNAs	*trnC(GCA), trnD(GUC), trnE(UUC), trnF(GAA), trnfM(CAU), trnG(GCC), trnI(CAU), trnL(CAA), trnL(UAG), trnM(CAU), trnN(GUU), trnP(UGG), trnQ(UUG), trnR(ACG), trnR(UCU), trnS(GGA), trnS(GCU), trnS(UGA), trnT(GGU), trnT(UGU), trnV(GAC), trnW(CCA), trnY(GUA)*

**Table 3:** Comparative Chloroplast Genomic Analysis in Santalales.

Species	*E. scandens*	*S. jasminodora*	*S. parasitica*	*T. chinensis*	*T. nigrans*	*T. sutchuenensis*	*C. manillana*	*O. alba*	*V. album*	*V. coloratum*	*V. crassulae*	*V. minimum*
Family	Erythropalaceae	Schoepfiaceae	Loranthaceae	Loranthaceae	Loranthaceae	Loranthaceae	Opiliaceae	Santalaceae	Santalaceae	Santalaceae	Santalaceae	Santalaceae
Accession No.	NC_036759.1	NC_034228.1	MH101514	NC_036306.1	MH095982	NC_036307.1	NC_034931.1	NC_027960.1	NC_028012.1	NC_035414.1	NC_027959.1	NC_027829.1
Genome size (bp)	156,154	118,743	121,750	121,363	121,419	122,562	147,461	147,253	128,921	128,744	126,064	131,016
LSC length (bp)	84,799	84,168	70,270	70,357	70,181	70,630	83,505	84,466	73,893	73,684	73,225	75,814
LSC length (%)	54.3	70.9	57.7	58.0	57.8	57.6	56.6	57.4	57.3	57.2	58.1	57.9
SSC length (bp)	18,567	9763	6106	6082	6100	6102	7806	13,972	8632	8630	8628	9014
SSC length (%)	11.9	8.2	5.0	5.0	5.0	5.0	5.3	9.5	6.7	6.7	6.8	6.9
IR length (bp)	26,394	12,406	22,687	22,462	22,569	22,915	28,075	24,340	23,198	23,215	22,105	23,094
IR length (%)	33.8	20.9	37.3	37.0	37.2	37.4	38.1	33.1	36.0	36.1	35.1	35.2
GC content (%)	38.0	38.1	37.2	37.3	37.4	37.3	37.4	37.7	36.4	36.3	36.4	36.2
Number of genes	130	121	106	106	106	106	120	117	115	119	116	104
Number of protein coding genes	86	72	66	66	66	66	73	74	71	70	73	66
Number of tRNAs	36	34	28	28	28	28	37	35	36	36	35	29
Number of rRNAs	8	8	8	8	8	8	8	8	8	8	8	8

**Table 4:** Codon–anticodon recognition patterns and codon usage of the *T. nigrans* and *S. parasitica* chloroplast genomes.

Species	Amino Acid	Codon	No.	RSCU	tRNA	Amino Acid	Codon	No.	RSCU	tRNA	Amino Acid	Codon	No.	RSCU	tRNA
*T. nigrans*	Phe	UUU	1821	1.22		Tyr	UAU	1227	1.38		Stop	UAG	584	0.75	
	Phe	UUC	1153	0.78	*trnF-GAA*	Tyr	UAC	545	0.62	*trnY-GUA*	Leu	UUG	863	1.3	*trnL-CAA*
	Leu	UUA	877	1.32		Stop	UAA	961	1.24		Leu	CUC	497	0.75	
	Leu	CUU	807	1.21		His	CAU	773	1.41		His	CAC	321	0.59	
	Leu	CUA	579	0.87	*trnL-UAG*	Gln	CAA	785	1.33	*trnQ-UUG*	Asn	AAC	600	0.59	*trnN-GUU*
	Leu	CUG	370	0.56		Gln	CAG	397	0.67		Asn	AAU	1419	1.41	
	Ile	AUU	1387	1.21		Ile	AUC	905	0.79		Ile	AUA	1148	1	
	Asp	GAU	789	1.44		Lys	AAA	1617	1.37		Lys	AAG	748	0.63	
	Met	AUG	726	1	*trn(f)M-CAU* *trnI-CAU*	Val	GUC	369	0.74	*trnV-GAC*	Asp	GAC	304	0.56	*trnD-GUC*
	Val	GUU	659	1.32		Val	GUA	599	1.2		Val	GUG	370	0.74	
	Ser	UCC	593	1.03	*trnS-GGA*	Glu	GAA	997	1.37	*trnE-UUC*	Cys	UGC	316	0.74	*trnC-GCA*
	Ser	UCU	816	1.42		Cys	UGU	535	1.26		Glu	GAG	455	0.63	
	Ser	UCG	490	0.85		Stop	UGA	777	1		Pro	CCU	536	1.08	
	Pro	CCC	499	1.01		Arg	CGC	228	0.56		Arg	CGA	461	1.14	
	Trp	UGG	581	1	*trnW-CCA*	Arg	CGU	267	0.66	*trnR-ACG*	Ser	UCA	684	1.19	*trnS-UGA*
	Pro	CCA	589	1.19	*trnP-UGG*	Thr	ACC	478	1.01	*trnT-GGU*	Ser	AGC	367	0.64	*trnS-GCU*
	Pro	CCG	358	0.72		Arg	CGG	301	0.75		Ser	AGU	491	0.86	
	Thr	ACU	532	1.13		Arg	AGG	466	1.15		Thr	ACG	331	0.7	
	Thr	ACA	545	1.16	*trnT-UGU*	Arg	AGA	700	1.73	*trnR-UCU*	Gly	GGC	267	0.63	*trnG-GCC*
	Ala	GCU	376	1.28		Ala	GCC	277	0.94		Ala	GCA	314	1.07	
	Ala	GCG	206	0.7		Gly	GGA	527	1.23		Gly	GGU	444	1.04	
	Gly	GGG	469	1.1											
*S. parasitica*	Phe	UUU	1842	1.23		Tyr	UAU	1157	1.33		Stop	UAA	985	1.32	
	Phe	UUC	1151	0.77	*trnF-GAA*	Tyr	UAC	588	0.67	*trnY-GUA*	Arg	AGA	730	1.76	*trnR-UCU*
	Leu	UUA	883	1.32		Stop	UAG	542	0.72		Leu	CUU	777	1.16	
	Leu	CUC	477	0.71		His	CAC	325	0.66		His	CAU	664	1.34	
	Leu	CUA	578	0.86	*trnL-UAG*	Gln	CAA	738	1.35	*trnQ-UUG*	Leu	UUG	909	1.35	*trnL-CAA*
	Leu	CUG	403	0.6		Gln	CAG	354	0.65		Ile	AUU	1468	1.27	
	Ile	AUC	781	0.67		Asn	AAU	1438	1.39		Lys	AAG	732	0.6	
	Met	AUG	701	1	*trn(f)M-CAU* *trnI-CAU*	Asn	AAC	624	0.61	*trnN-GUU*	Glu	GAA	941	1.34	*trnE-UUC*
	Ile	AUA	1223	1.06		Lys	AAA	1725	1.4		Val	GUA	571	1.19	
	Val	GUU	673	1.4		Asp	GAU	832	1.4		Val	GUG	350	0.73	
	Val	GUC	328	0.68	*trnV-GAC*	Asp	GAC	360	0.6	*trnD-GUC*	Ser	UCA	709	1.15	*trnS-UGA*
	Ser	UCU	861	1.4		Cys	UGU	603	1.28		Glu	GAG	468	0.66	
	Ser	UCC	685	1.11	*trnS-GGA*	Cys	UGC	336	0.72	*trnC-GCA*	Trp	UGG	524	1	*trnW-CCA*
	Ser	UCG	508	0.83		Stop	UGA	716	0.96		Arg	CGA	457	1.1	
	Pro	CCU	480	1.01		Arg	CGU	305	0.74	*trnR-ACG*	Pro	CCA	569	1.2	*trnP-UGG*
	Pro	CCC	519	1.1		Pro	CCG	324	0.68		Arg	CGC	217	0.52	
	Thr	ACU	510	1.11		Ser	AGU	540	0.88		Arg	CGG	317	0.77	
	Thr	ACC	470	1.03	*trnT-GGU*	Ser	AGC	388	0.63	*trnS-GCU*	Gly	GGC	278	0.65	*trnG-GCC*
	Thr	ACA	530	1.16	*trnT-UGU*	Ala	GCC	279	0.94		Arg	AGG	460	1.11	
	Thr	ACG	321	0.7		Ala	GCG	186	0.62		Ala	GCA	352	1.18	
	Ala	GCU	374	1.26		Gly	GGA	563	1.31		Gly	GGU	421	0.98	
	Gly	GGG	462	1.07											

**Table 5:** Genes with introns in the *T. nigrans* and *S. parasitica* chloroplast genomes, including the exon and intron length.

Species	Gene	Location	Exon I(bp)	Intron I(bp)	Exon II(bp)	Intron II(bp)	Exon III(bp)
*T. nigrans*	*atpF*	LSC	150	786	390		
	*rpoC1*	LSC	450	756	1602		
	*ycf3*	LSC	127	755	230	785	153
	rps12 *	LSC, IR	114	-	232	543	26
	*petB*	LSC	6	798	642		
	*petD*	LSC	9	696	483		
	*rpl2*	LSC, IR	394	652	434		
*S. parasitica*	*atpF*	LSC	150	759	390		
	*rpoC1*	LSC	456	778	1626		
	*ycf3*	LSC	153	712	230	759	127
	*petB*	LSC	6	700	642		
	*petD*	LSC	9	652	483		
	*rpl2*	LSC, IR	394	665	431		
	rps12 *	LSC, IR	114	-	232	534	26

* The *rps12* gene is divided into 5’-*rps12* in the LSC region and 3’-*rps12* in the IR region.

## Data Availability

The data presented in this study are available upon request from the corresponding author.
